# The influence of the mating type on virulence of *Mucor irregularis*

**DOI:** 10.1038/s41598-017-10954-2

**Published:** 2017-09-06

**Authors:** Wenqi Xu, Guanzhao Liang, Jingwen Peng, Zhimin Long, Dongmei Li, Meihua Fu, Qiong Wang, Yongnian Shen, Guixia Lv, Huan Mei, Clement K. M. Tsui, Weida Liu

**Affiliations:** 1 Department of Mycology, Institute of Dermatology, Chinese Academy of Medical, Sciences and Peking Union Medical College, No. 12 Jiang Wangmiao Street, Nanjing, 210042 Jiangsu People’s Republic of China; 2 Demo Lab, Shanghai AB Sciex Analytical Instrument Trading Co., Ltd, North Fuquan Road, IBP, Shanghai, 200335 People’s Republic of China; 3 Department of Microbiology and Immunology, Georgetown University Medical Center, Washington DC, 20057 USA; 4 Division of Infectious Diseases, Faculty of Medicine, University of British Columbia, Vancouver, BC V6H 3Z6 Canada

## Abstract

*Mucor irregularis* is an emerging fungal pathogen that cause cutaneous infection and could cause death. However, little is known about its mechanism of pathogenesis. There is evidence suggesting virulence vary with mating types in fungi, including the Mucorales. Here, we characterized the mating type locus of *M*. *irregularis* and the mating type ratio of 17 clinical isolates in China. Genomic data indicated *M*. *irregularis* is heterothallic having two mating types – bearing either *SexP* or *SexM* allele. Also, we employed a mice model to study the inflammation and pathological effects of different mating types. The comparison of the inflammatory response, cytokine profiles and Th-1, Th-2 and Th-17 cells numbers in each mating type treated mice showed that the severity and disease progress were enhanced in (+) mating type treated mice. One (+/0) mutant strain, with multiple mutations at the mating locus, had defects in sexual mating ability but appeared to be more virulent than the (−) mating type. Although (+) mating type appeared to be more virulent, most of our clinical isolates presented belonged to (−) mating type. Our findings support the involvement of *MAT* genes in sexual fertility, and the influence of mating type on the severity of cutaneous infection.

## Introduction

Mucormycosis is an invasive fungal infection that almost invariably occurs in immunocompromised patients and it is the third most common life-threatening fungal infection following aspergillosis and candidiasis^1^. Mucormycosis is usually caused by a group of angioinvasive fungi in the order of Mucorales. *Mucor irregularis* Stchigel, Cano, Guarro & E. Álvarez (synonym: *Rhizomucor variabilis* R.Y. Zheng & G.Q. Chen), a causative agent of mucormycosis, was firstly reported in 1991, on the hand of a woman in China with a primary cutaneous infection^2^. Since then, dozens of *M*. *irregularis* infection have been reported in China^3–6^, India^7, 8^, Japan^9, 10^, USA^11, 12^, Australia^13^ and France^14^. Mucormycosis caused by *M*. *irregularis* are characterized by progressive central facial swelling, ulceration and midline facial destruction with pathological features of inflammation, necrosis, ultimately leading to severe disfigurement and even death if left untreated^4, 5, 15^ (see Supplementary Fig. S1). However, little is known about the pathogenic mechanism of *M*. *irregularis* that is critically important for the prevention and treatment of mucormycosis.

Sexual reproduction is ubiquitous across fungi and important to the fitness of species. It has been noted that sexual reproduction coupled with meiosis and recombination plays an important role in the evolution of pathogenic fungi and could have significance influence on virulence^16^. For any fungus that can reproduce through a sexual cycle, different mating types are required to form sexual/gametes fusion^17^. In fungi, the mating process is controlled by a sex-specific region of the genome known as the mating type locus (*MAT*), and the *MAT* loci determination in fungi has predominantly been studied in ascomycetes and basidiomycetes. However, sexual reproduction has not been demonstrated in the Mucorales until recently, for example, *Rhizopus miehei*, *Mucor circinelloides*, *Phycomyces blakesleeanus*, *Rhizopus oryzae* and other human pathogenic members of the Mucorales^18–20^. These studies revealed that the mating system in Mucorales were regulated by divergent alleles of a single gene - *SexM*/*SexP*, which consist of a high mobility group (HMG) transcription factor gene flanked by genes encoding a triose phosphate transporter homolog (TPT) and an RNA helicase^21–23^. The HMG domain proteins are designated as SexP for the (+) and SexM for the (−) mating types respectively. The sequences of the genes encoding SexP and SexM are divergent but bi-allelic at the *MAT* loci, in contrast to the idiomorphic nature of *MAT* loci in many ascomycetes and basidiomycetes that encoding entirely divergent proteins^24^.

The process of sexual reproduction in the fungi has been linked to the virulence and the outbreak of infectious diseases. For instance, the mating type has been found to be a source of variation in virulence in some opportunistic fungal pathogens, such as *A*. *fumigatus* ^25^. Studies have demonstrated a predominance of *MAT1-1* isolates among invasive aspergillosis cases, as well *MAT1-1* isolates in the larvae of *Galleria mellonella* were more virulent than *MAT1-2* isolates^25, 26^. While in *Cryptococcus neoformans*, the α mating-type isolates have greater abilities to cause virulence than **a** mating type^27^. Even the *MAT* locus genes themselves have been associated with the pathogenesis of *Fusarium graminearum*, as demonstrated by the reduced virulence of mat1-1-1 and mat1-2-1 mutants in corn stalk rot assays^28^.

In Mucorales, increased pathogenicity in (+) mating types has been observed in pathogenic fungi. For example, when spore suspensions of plant pathogenic *Mucor piriformis* were used as inoculum, the (+) mating type produced significantly larger lesions than those produced by the (−) type^29^. Similarly Stewart and Munday^30^ found that the (+) mating type of *M*. *amphibiorum* may be capable of causing greater rates and severe infection in toads than the (−) mating type. The (+) mating type produced spherules more rapidly with daughter cells, leading to a more rapid dissemination of infective propagules and a more severe infection^30^. In contrast, the (−) mating type isolates of *Mucor circinelloides f*. *lusitanicus* (Mcl) were more virulent in the wax moth host compared to (+) isolates; however, the loss of virulence in (−) mating type was associated with the sporangiospore size dimorphism, not related directly to the *MAT* locus^31, 32^. Nevertheless, a sexual cycle has never been studied in *M*. *irregularis*, although genetic variation has been reported among isolates^15^. The relationship between genetic/genomic variation and the level of virulence is also poorly investigated.

In this study, we hypothesized the virulence of *M*. *irregularis* can vary with its mating types. First, we characterized the *MAT* locus and the *SexM/P* genes of *M*. *irregularis* using genomic and morphological methods. Second, we analyzed the mating type of 17 clinical isolates that were collected from China. All isolates, but the standard strain CBS103.93 (−) have not been characterized in previous investigation^12^. Third, we investigated the impact of mating type on fungal virulence by using a subcutaneous injection mice model; we studied the virulence of three *M*. *irregularis* isolates with different mating types: B50k (+), B50n (+/0), and CBS103.93 (−) *in vivo*. Through the comparison of pathological alternation under microscopy, serum cytokine profiles and the percentages of T helper 1 (Th-1), Th-2 and Th-17 cells among three isolates in the murine model, we found that the severities of inflammation and immunoreaction were more prevalent in *M*. *irregularis* (+) mating type. The findings from *in vivo* study suggested the two mating types of *M*. *irregularis* may have different impact on virulence phenotypes and clinical manifestations.

## Results

### Morphological and physiological features

**Figure 1:**
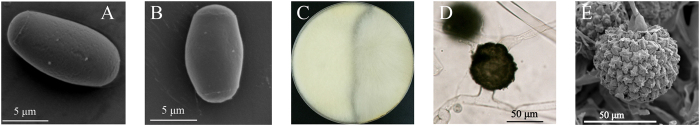
Zygospores formed in the intraspecific cross of *Mucor irregularis* CBS103.93 (−) × B50k (+). (**A**,**B**) SEM images of CBS103.93 and B50k spores. The surface of two mating type isolates are both smooth. (**C**) Line of zygospores formed on MEA 10 days after CBS103.93 × B50k were paired. (**D**) Electron micrograph of a cross between CBS103.93 × B50k showing zygospores; bar, 50 μm. (**E**) SEM image of mature zygospores with stellate ornamentation; bar, 50 μm.

All the *M*. *irregularis* isolates (CBS103.93 and CMFCCC B50e~B50t, Table S1) produced whitish to yellowish colonies that were yellow on the reverse when grown on MEA. The sporangiospores were hyaline, smooth walled (Fig. 1A,B), variable in size and shape, sub-spherical to ellipsoidal or reniform, ca. 3.7–13.3 × 2.0–10.3 μm. We found the growth rates and the optimal growth temperatures of clinical isolates were similar to the standard strain on MEA at room temperature (~21 °C), 24 °C, 28 °C, 32 °C, 35 °C, and 38 °C. After 4 days, colonies had grown over the plates at 24 °C, 28 °C, 32 °C, except at 35 °C. No growth was observed at 38 °C after 3 weeks in any isolates. The inability to grow at temperatures >37 °C was consistent to previous reports^2^.

### Mating type determination through crossing experiment

After 10 days incubation at 25 °C in the dark on MEA, zygospores was typically formed in a prominent dark band at the contact zone between *M*. *irregularis* CBS 103.93 (−) and (+) isolates such as B50k and B50r as shown in Fig. 1C. The typical zygospores of *M*. *irregularis* were medium to dark brown (Fig. 1D), up to about 60 μm in diameter, with stellate ornamentations (Fig. 1E) and opposed suspensors. No sexual reactivity was observed when CBS 103.93 were pairing with 14 *M*. *irregularis* isolates, which in contrast can form zygospores while crossing with B50k (+). Therefore, these 14 isolates were presumably to be (−) type, the same mating type as CBS103.93.

### Dissimilarity between *SexP* and *SexM* at the MAT locus

**Figure 2:**
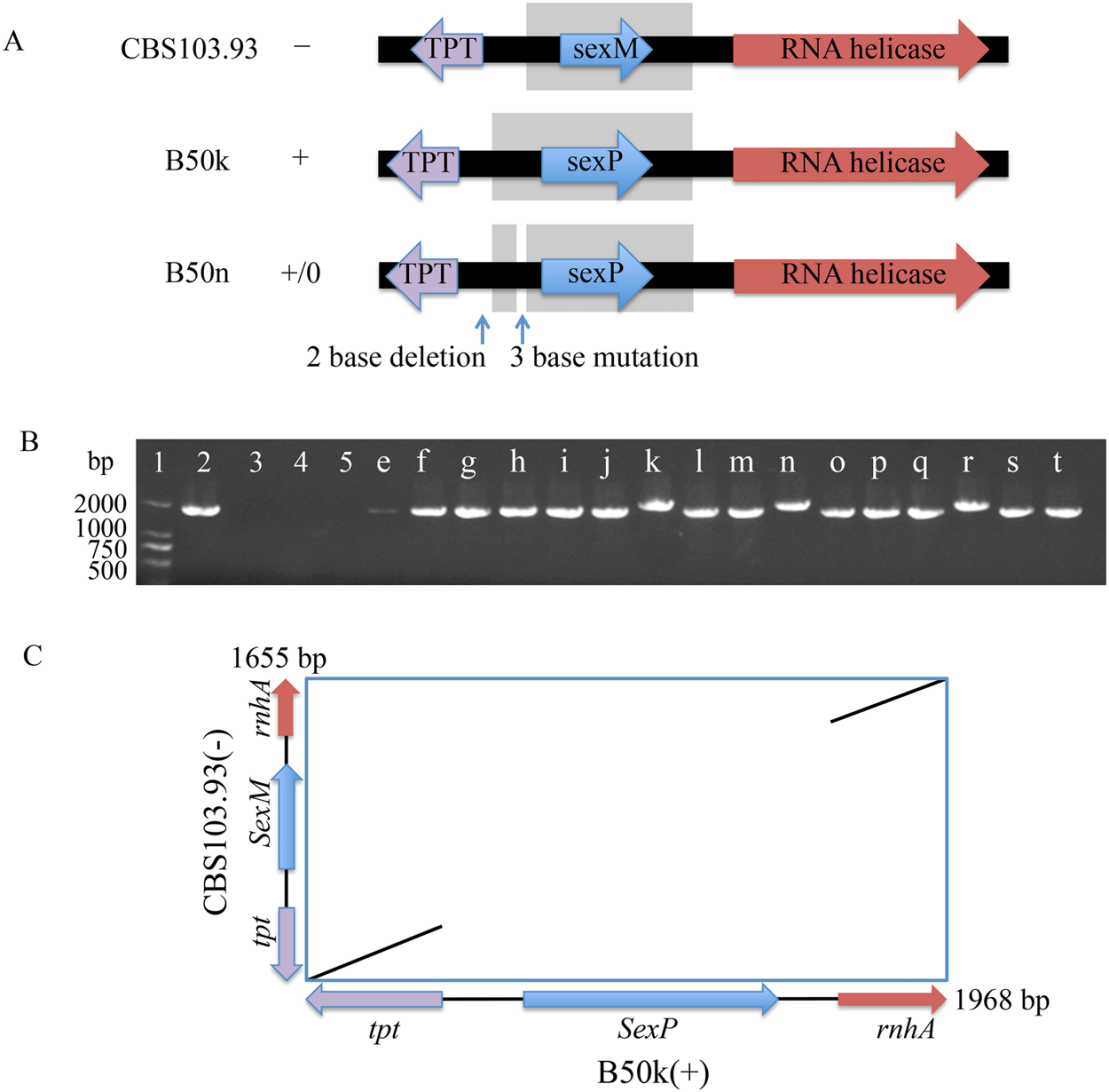
Sequence analysis of the MAT locus in *Mucor irregularis*. **(A**) Arrangement of *MAT* locus for the SexM (−) or SexP (+) alleles with their adjacent genes in *M*. *irregularis*. The *SexM* and *SexP* in *M*. *irregularis* are flanked by genes for RNA helicase and a triosephosphate transporter. The orientations of *SexP* and *SexM* genes are the same. The dissimilar DNA sequences of the *sexM/P* (from the TSS to stop codon) of *M*. *irregularis* are highlighted by a light grey box, gene orientation are show in the arrows. (**B**) Mating-type determination of *M*. *irregularis* isolates by PCR. DNA amplified with the *MAT* specific primer designed from5’ of *tpt* to the 5’ of *RNA helicase*. Lane 1: molecular ladder; Lane 2: *M*. *irregularis* CBS103.93; lane 3: no template control; lane 4: ddH_2_O (as the negative control); lane 5: *Rhizopus oryzae* B81a (as the negative control); lanes e-t: *M*. *irregularis* isolates B50e- B50t. (**C**) Dot plot comparison between the homeodomain region of *M*. *irregularis* isolates CMFCCC B50k (x-axis) and CBS103.93 (y-axis).

The *MAT* locus in *M*. *irregularis* B50p genome (accession No. AZYI00000000.1.) was identified by using the top hits from TBLASTN queried with the known *MAT* locus sequences in from other members of Mucorales. The gene organization at the locus was predicted using FGENESH pipeline (http://www.softberry.com/berry.phtml) and was searched against the genome sequences of the *R*. *oryzae*, *M*. *circinelloides*, and *P*. *blakesleeanus* genome databases in the GenBank. Genomic analysis showed that *M*. *irregularis* isolate B50p contained a single *SexM* gene with HMG domain, flanked by a predicted triose phosphate transporter (*tpt*) and RNA helicase (*rnhA*) genes (Fig. 2A).

To examine whether the *MAT* locus was identical in sequences among all the SexP (+) and SexM (−) mating types of *M*. *irregularis* isolates, PCR was performed using a specific primer pair targeting from the 5′ of *tpt* to the 5’ of *rnhA* of B50p. A single band was amplified from all isolates, and the size of fragments was either 1660 or 1960 bp (Fig. 2B). The sequenced results demonstrated that the (+) type had longer fragment, and the (−) type isolates had shorter fragment with the exception of B50n, which had the same longer band as the (+) type. Every isolate contains a single gene, either *SexM* or *SexP*, at the locus flanked by the genes *tpt* and *rnhA*, respectively (Fig. 2A). The overall ratio of SexM (−): SexP (+) in China was 14:3.

DNA dot-plot analysis between the homeodomain region of CBS103.93 (−) and the corresponding region from B50k (+) showed that there was a highly dissimilar core corresponding to *SexM* and *SexP* genes (only 32.4% similarity in nucleotide); in contrast the *tpt* and *rnhA* were highly similar between two mating types (99.4% and 97.3% similarities, respectively) (Fig. 2C).

The *SexP* gene was 933 bp long and had no intron. A transcription start site (TSS) was located 157 bp upstream of the start codon. Comparison of the promoter region (264 bp upstream of the start codon) of *SexP* from *M*. *irregularis* with sequences of other Mucorales revealed a conserved transcription factor binding motif with a CCAAT-box (GATCxxxxAxCCAAT) located at 73 bp upstream of the start codon. *SexM* (579 bp long) was shorter than *SexP*, no intron and shared only 32.4% DNA sequence identity with *SexP*. Also a TSS was found at location of 195 bp upstream of the start codon. Comparing the promoter regions in *SexM* (359 bp upstream of the start codon) between *M*. *irregularis* and other Mucorales did not reveal any conserved sequences. Similarly, there was no conserved sequence between the promoter regions of *SexM* and *SexP* in *M*. *irregularis*, indicating that both genes may be regulated by different mechanisms and processes.

The amino acid sequences similarities of SexP and SexM to other Mucoralean fungi ranged from 25 to 60%, and from 26 to 36%, respectively. The SexP in *M*. *irregularis* had the greatest similarity to SexP in *M*. *mucedo*, and phylogenetic analysis showed that the SexP proteins of *M*. *irregularis* and *M*. *mucedo* clustered together (see Supplementary Fig. S2). Unexpectedly, although the *M*. *irregularis* SexM shared the highest similarity with SexM of *R*. *delemar*, phylogenetic analysis indicated that SexM of *M*. *irregularis* was grouped within the SexP subgroup (see Supplementary Fig. S2), and distinct from all other SexM proteins, suggesting the SexM/P of *M*. *irregularis* may be evolved before speciation within the Mucorales.

Comparative analysis of these *MAT* locus sequences indicated that SexP (+) type was differentiated from SexM (−) type, however the intraspecific variation between SexM/P sequences was quite low among each type. Moreover, the phylogenetic tree showed that the distribution of mating type, at least the SexM (−) type, might have little correlation to the source of isolates in China (see Supplementary Fig. S3 and Table S1).

**Figure 3:**
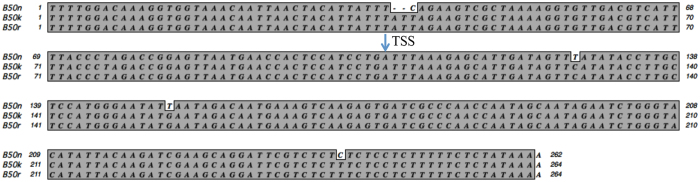
Alignments of the promoter region of *SexP* among three SexP (+) type *Mucor irregularis* isolates. The multiple alignments were performed using the ClustalW program implemented in MacVector 11.

Of all the isolates, B50n appeared to be a natural mutant and was labeled as (+/0). First, although the size of its *MAT* locus belonged to the (+) type, it could not mate with CBS103.93 (−). Second, there was a 2 bp deletion (at the upstream of TSS) and 4 mutations at the upstream region of *SexP* start codon in this isolate in comparison to those of B50k and B50r (Fig. 3). Third, when B50n was paired with other 16 isolates, only 5 (−) isolates (B50f, o, p, q and t) presented mating responses; it failed to form zygospores when crossed with other (+) or (−) types isolates, indicating that B50n was partially defect in sexual reproduction.

### Mice were susceptible to *M*. irregularis infection

To elucidate the possible variation in the pathogenicity among the different mating types of *M*. *irregularis*, a mice model was employed. Subcutaneous injection model in the back of BALB/c mice was used as an indicator of the degree of inflammation. Three isolates of various mating types and clinical manifestation profiles were selected: the standard strain CBS103.93 (−), the natural *SexP* mutant isolate B50n (+/0) and B50k (+), which was isolated from a patient who developed severe clinical symptoms (unpublished data, Fig. S1). *R*. *oryze* B81a was used as a positive control due to its aggressive pathogenicity and the saline (0.9% NaCl) was used as the negative control.

**Figure 4:**
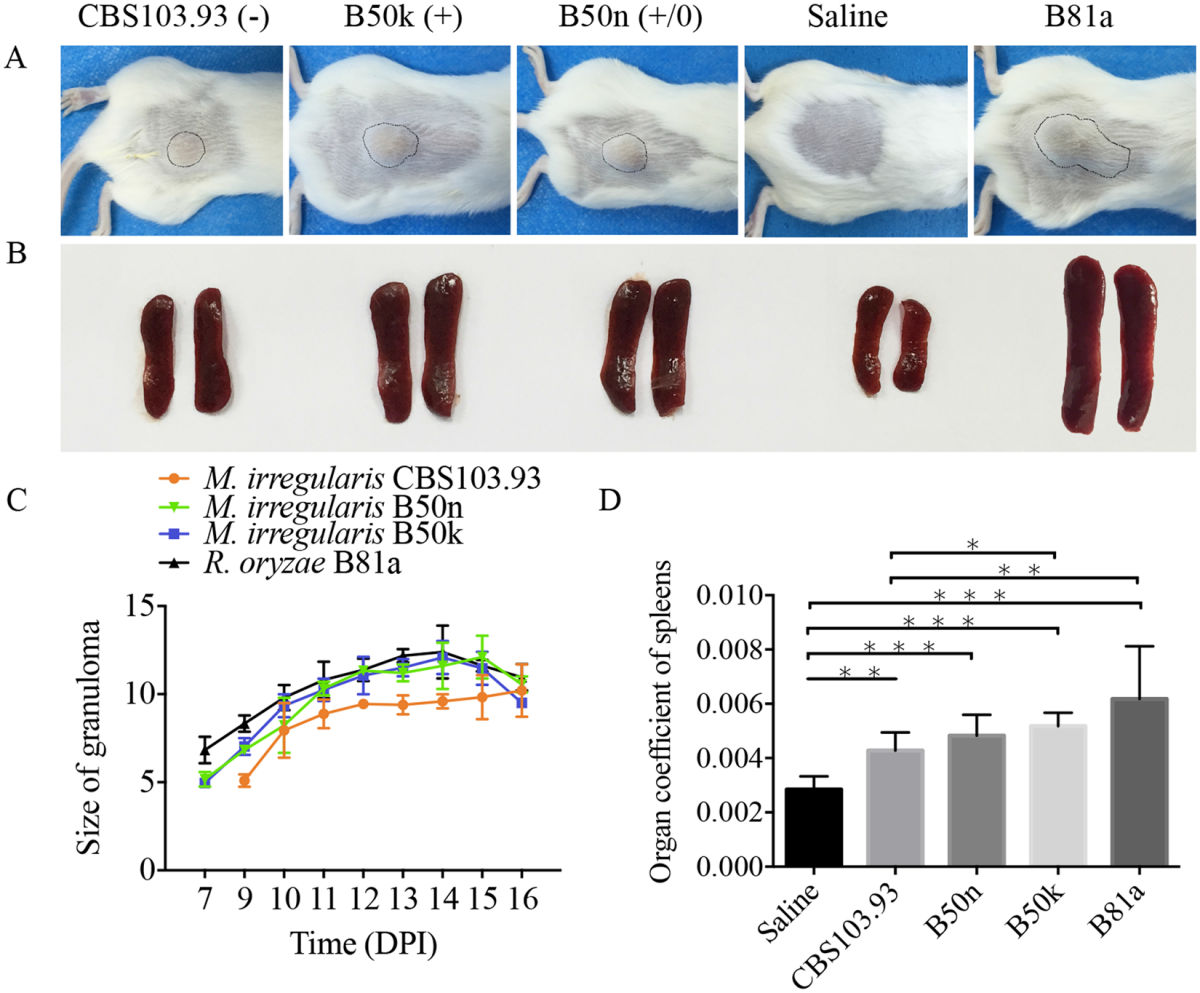
Clinical progression of mice model induced by intradermal injection of *Mucor irregularis* CBS103.93, B50n and B50k; *Rhizopus oryzae* B81a was used as a positive control and the saline was the negative control. (**A**) Representative clinical presentation at 15 days post infection (lumps in circles). (**B**) Demonstration of splenomegaly in mice injected with different isolates. Groups of BALB/c mice were injected with each isolate, and after 15 days their spleens were sacrificed for observation. (**C)** The lump development following intradermal infection. Data are shown with mean ± SD (*n* = 20). (**D**) Effects of injection from four Mucorales on organ coefficients of the mice spleens. Organ coefficient was calculated as the percentage of the ratio of wet weight of the spleens to the body weight. The data represents means ± SD. *p < 0.05, **p < 0.01, and ***p < 0.001 was considered statistically significant.

**Figure 5:**
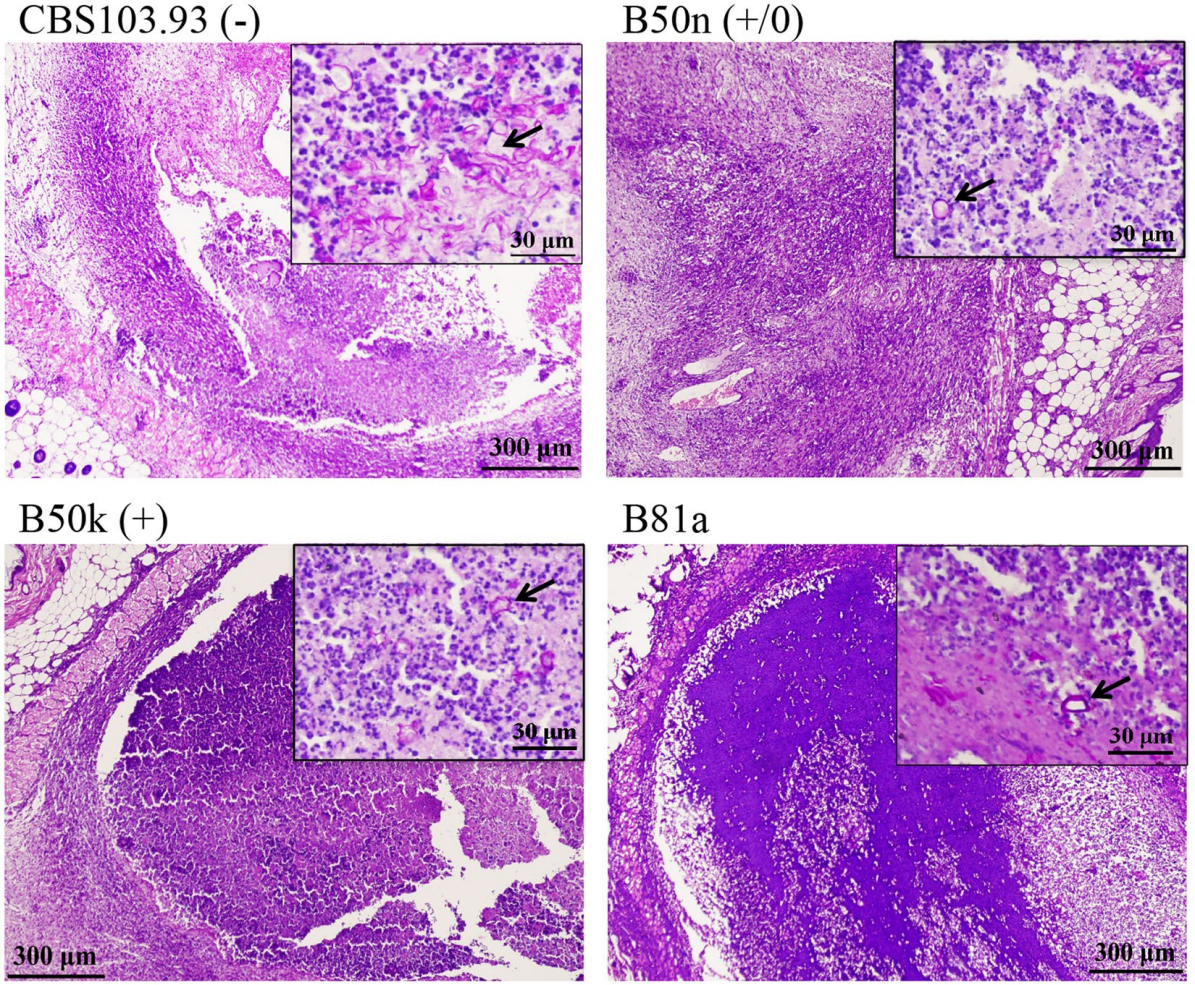
Photomicrography of the dermis and cutis of BALB/c mice experimentally infected with *Mucor irregularis* CBS103.93, B50n, B50k and *Rhizopus oryzae* B81a at 15 days after the injection. HE staining of dermis sections (Low-power sections, 100X, bars = 300 μm) revealed the presence of an accentuated inflammatory infiltrate. PAS staining (upper right corner of each graph, high power magnification, 400X, bars = 30 μm) revealed the presence of hypha (arrow).

Seven days after the inoculation, a small lump in BALB/c mice was observed (Fig. 4C). After 14 days, B50k (+)-injected mice presented much larger lump (*p* = 0.0258) than CBS103.93 (−) (Fig. 4A,C) and more serious splenomegaly (Fig. 4B,D), indicating a more serious immunoreaction. The lump in B50n (+/0)-injected group was also larger than CBS103.03 group, but smaller than the B50k (+) group. In general, the severest manifestation of the lesions in the model mice was observed at about 15 days (Fig. 4C). To further analyze the degree of inflammation and pathological characteristics in BALB/c mice, the lesion was biopsied at 15 day after infection (DAI). Under the microscopic examination, a main feature of the lesion including inflammation, necrosis, and granulation was evident, along with a non-specific pattern of inflammatory response infiltrated with neutrophil granulocytes, lymphocytes, plasma cells, and sometimes multinucleated macrophages (Fig. 5). Consistent with these general observation, B50k (+)-injected group presented more serious inflammatory cell infiltration and necrosis than CBS103.93 (−) -injected group (Fig. 5), suggesting an enhanced immune response in B50k (+) group. B50n (+/0)-injected group had mediate inflammatory reaction between those of B50k (+) and CBS103.93 (−) (Fig. 5).

### Differential Th-1, Th-2 and Th-17 cytokine profiles after *M*. *irregularis* challenge

**Figure 6:**
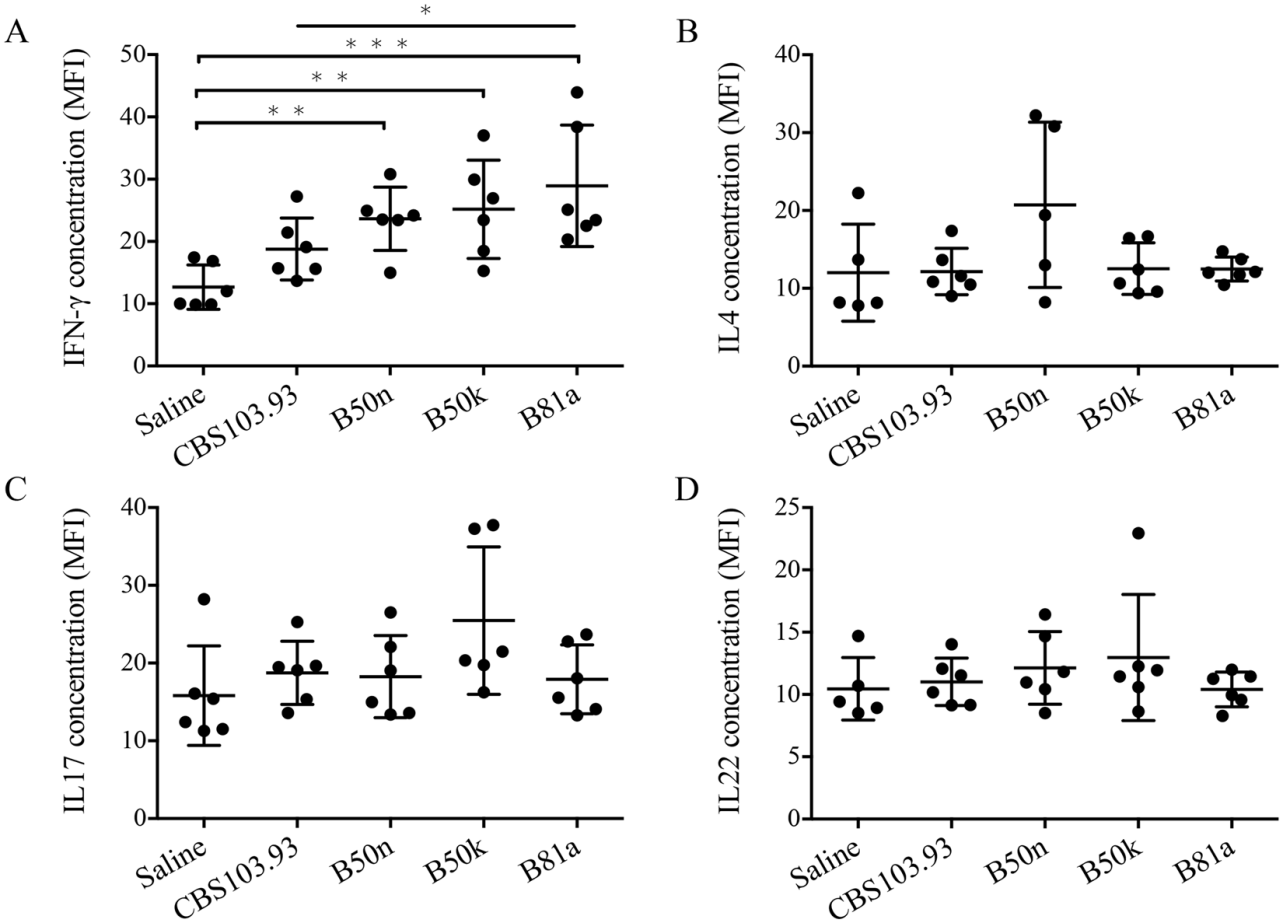
Effects of injection from four Mucorales on cytokine levels in serum. Serum IFN-γ (**A**), IL-4 (**B**), IL-17 (**C**) and IL-22 (**D**) concentrations were measured at 15 days after the injection of *Mucor irregularis* CBS103.93, B50n, B50k and *Rhizopus oryzae* B81a. Saline-treated mice served as control.

The host defense mechanisms against fungi usually range from an early non-specific immune response to activation and induction of specific adaptive immunoreaction by the production of Th-1, Th-2 and Th-17 cytokines. To determine the levels of Th-1, Th-2 and Th-17 cytokines induced in mice infected with *M*. *irregularis*, and the association between their levels and disease prognosis, numerous cytokines in serum was examined by Luminex. Higher levels of the Th-1 cytokines (IFN-γ) were found in B50k-injected mice than CBS103.93-injected ones (Fig. 6A), while *R*. *oryze* B81a group (positive control) had the highest level among all groups. Again, B50n group still displayed higher level of cytokines than CBS103.93. However, the Th-2 cytokines IL-4 (Fig. 6B) showed no significant difference among groups. The levels of Th-17 cytokines (IL-17, IL-22) showed minor difference (Fig. 6C,D), even though the average level of B50k group was higher than that in B50n and CBS103.93 groups. However, other cytokines such as IL-1α, IL-1β, MCP-1, M-CSF (CSF-1), MCP-3, MIP-1α, MIP-1β, MIP-2, TNFα were not significantly different among tested groups.

**Figure 7:**
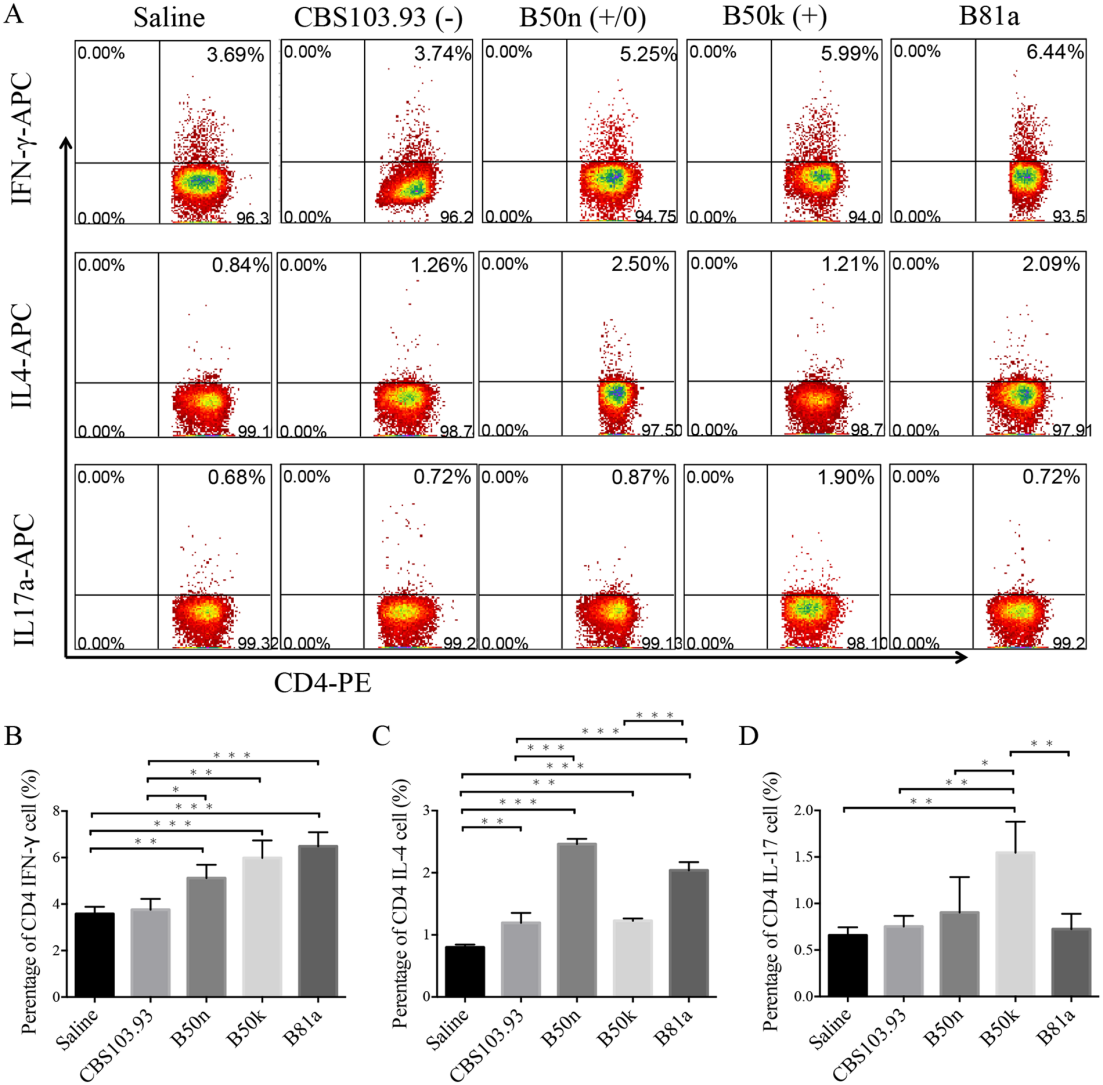
Effects of injection from four Mucoralean fungi on the proportion of Th cells in the spleen. Original dot plots from FACS analysis of CD4^+^ IFN-γ^+^ cells (**A**,**B**), CD4^+^ IL4^+^ cells (**A**,**C**), and CD4^+^ IL17^+^ cells (**A**,**D**) isolated from spleens. The numbers in the dot plots indicated the percentage of cells whining each quadrant. B, C and D showed the percentage of CD4^+^ IFN-γ^+^ cells, CD4^+^ IL4^+^ cells, and CD4^+^ IL17^+^ cells of the splenocytes. Data are mean ± SD of five mice, in two separate experiments. *p < 0.05, **p < 0.01, and ***p < 0.001.

Guided by the cytokine profiles, Th-1, Th-2 and Th-17 cells numbers in mice of each group was evaluated by flow cytometry. Percentage of Th-1 cell (CD4^+^IFN-γ^+^) in B50k group (5.98 ± 0.70%) was significantly higher than that in CBS103.93 group (3.58 ± 0.34%) (Fig. 7A,B), but lower than that of *R*. *oryze* B81a group (6.48 ± 0.58%). The Th-1 cell percentage of B50n group (5.11 ± 0.57%) was also higher than CBS103.93 but lower than B50k (Fig. 7A,B). Similar to the serum cytokine profiles, percentage of Th-2 cells (CD4^+^IL-4^+^) in B50k group (1.22 ± 0.04%) did not differ from those in CBS103.93 group (1.19 ± 0.16%), while B50n mutant group (2.46 ± 0.09%) showed significantly higher percentage (~1 fold increase) than CBS103.93 and B50k (Fig. 7A,C). Notably, B50k group showed highest percentage of Th-17 (CD4^+^IL-17^+^) (1.55 ± 0.33%) when compared to control (0.67 ± 0.09%), CBS103.93 (0.75 ± 0.11%), and B50n (0.90 ± 0.38%) (Fig. 7A,D).

## Discussion

*Mucor irregularis* has emerged as a lethal infectious disease to the human hosts^10–15^. From the clinical point view, immunodeficiency or immunosuppression may not account for all the etiology and pathogenesis of the *M*. *irregularis* infection since *M*. *irregularis* infections are always occur in the patients without any apparent immune impairment, of which is different with other pathogenic fungi in Mucorales. Although, the pathogenic mechanism of *M*. *irregularis* remains largely unknown, increasing signs/evidence suggest the invasiveness or virulence of *M*. *irregularis* is closely related to the pathogenic process, independent to the biological variation of patients^15^. The genomic data of *M*. *irregularis* therefore becomes an important resource to seek for specific pathogenic mechanisms of *M*. *irregularis*.

The mating type has previously been linked to increased virulence in fungi^25–31^. In order to understand the possible correlation between mating type and virulence in *M*. *irregularis*, we characterized the mating type genes of 17 clinical isolates of *M*. *irregularis* that have been mostly collected from individual infected patients in China. The sequence data, coupled with the mating crosses results test showed that 14 clinical isolates are (−) type. The sequence data also demonstrated that each isolate possesses a mating type locus that is homologous to those of other Mucorales. Either SexM or SexP domain gene was identified in the *MAT* locus, demonstrating that *M*. *irregularis* is heterothallic. Although the *SexP* or *SexM* contained specific sequences that did not exist in the complementary mating type, both genes’ cluster consisted of an HMG domain gene flanked by *tpt* and RNA helicase genes, which located at the same chromosomal positions. Similar architecture of the putative mating type locus has also been found in other heterothallic Mucoralean fungi such as *M*. *circinelloides* ^21^ and *R*. *oryzae* ^23^. Together, the data suggested the *MAT* gene organization in Mucorales was ancient and conserved. However, unlike the *MAT* locus of *R*. *oryzae* which contained all genes in the *MAT* locus positioned at the same orientation, *TPT* and *SexM/P* genes in *M*. *irregularis* were in inverted orientations, which was the same as *P*. *blakesleeanus* and *M*. *circinelloides* ^21–23^.

Moreover, one isolate B50n with deleted/mutated *SexP* homolog at *SexP* mating locus, was identified in our study - the kind of natural mutation has never been reported previously in other heterothallic Mucorales. When the *SexP* has mutations, only 5 of the 14 SexM (−) isoates could mate with it. The partial defect in mating abilities in B50n mutant provided evidence that this *MAT* locus was involved in the regulation of mating process/sexual reproduction in *M*. *irregularis*. Similar observations have been reported in other fungi, for example, disrupting the *SexM* gene at the (−) mating type caused sterility in the (−) mating type of *M*. *circinelloides* ^32^; the *SsΔMAT-1b S*. *scitamineum* mutants were defective in mating when mixed with the opposite mating type^33^. Generally, the MAT locus has been corroborated across Mucorales species such as *R*. *oryzae*, *M*. *mucedo*, *M*. *circinelloides*, *P*. *blakesleeanus* and *M*. *irregularis*, indicating that the *MAT* locus governs sexual reproduction of the Mucorales.

We investigated the influence of mating type on the virulence of *M*. *irregularis*. Three *M*. *irregularis* isolates, ex-type CBS103.93 (−), B50n (+/0) and B50k (+) were used to compare their virulence through the *in vivo* murine model. Macroscopic and microcosmic analyses of the infected mice showed disease progression among the mating types; B50k (+) produced greater lesion and caused more damage to murine host than CBS103.93 (−). The murine data was consistent with the severity of clinical symptoms in patients (see Supplementary Fig. S1). Our findings indicated an association between mating type and virulence in *M*. *irregularis*; the (+) mating type appeared to be more virulent than the (−) mating type.

Contrasting virulence level between opposite mating types has been reported in other human fungal pathogens such as *Candida parapsilosis* and *Cryptococcus neoformans* ^34–38^. For example, Kwon-Chung^39^ demonstrated that the α mating type was more virulent than the **a** mating type, using congenic strains of *Cryptococcus neoformans* var. *neoformans*. Although, no difference in virulence between mating types has been found with *Cryptococcus neoformans* var. *grubii*, cells of α mating type have been shown to have an enhanced predilection to penetrate the central nervous system during co-infection experiments in mice^40^. Also in *M*. *circinelloides*, another member of Mucorales, (−) mating type isolates produced larger asexual sporangiospores that were more virulent in the wax moth host compared to (+) isolates that produce smaller less virulent sporangiospores^32^, and the greater virulence was related with the sporangiospore size for the larger spores could start invasive hyphal growth immediately upon phagocytosis by host immune cells, whereas smaller spores have a long period of isotropic growth^32^. Moreover, SEM analyses revealed that the larger (−) spores are decorated with ‘bumps’ on the surface, and the surface of the smaller (+) spores tended to be smooth in *M*. *circinelloides* ^32^. Unlike *M*. *circinelloides*, the surfaces of spores from both two mating types of *M*. *irregularis* were smooth, and there was no significant variation in the size of *M*. *irregularis* spores between two mating types, suggesting that spore size may be unrelated to the virulence in *M*. *irregularis*.

Apart from the variation in virulence between the two mating types of *M*. *irregularis*, the host immune responses were also different during the *M*. *irregularis* infection. At 15 DPI, B50k−injected BALB/c mice presented a significantly larger lump when compared to CBS103.93 mice, indicating more serious inflammation. Previous analysis of fungal infection has revealed that immunoreactions in the fungi−infected human or animal models were closely correlated with the invasiveness or virulence of fungi, and the immune mechanisms of defense against fungal infections are numerous, ranging from innate immunity to sophisticated adaptive mechanisms^41^. The Th-1, Th-2, and Th-17 cells (three major T helper cell subsets differentiated from CD4^+^ T cells) are crucial component of the adaptive immune response to fungal pathogens and the essential role in protecting body from being infected with diverse fungal pathogens^42^. Increased IFN-γ and IL-17 level in the serum, as well as an enhanced percent of CD4^+^IFN-γ^+^ (Th-1) and CD4^+^IL-17^+^ (Th-17) cells in the spleen of infected *M*. *irregularis* mice, suggested that cell-mediated immunity can play an important role in the pathogenesis of *M*. *irregularis* infection. In agreement of severity of inflammatory response, both cytokine profiles and Th-1 and Th-17 cell populations were highly responded in B50k-injected BALB/c mice. On the other hand, B50n (+/0), with a defective mating ability showed an intermediate inflammatory response and immune response between CBS103.93 and B50k, also suggested a can’t-be-ignored correlation between mating type and virulent level. From the results, we may conclude 3 points: first, cell-mediated immunity is playing an important role to control the cutaneous *M*. *irregularis* infection, especially the Th-1 and Th-17 pathway, consistent with our previous study that IL-17 and IFN-γ mRNA expression was significantly increased over time in *M*. *irregularis* infected skin lesion^43^; second, the mating type may have a potential correlation with the severity of inflammation; third, although the mating type gene is not a direct virulence factor, but link to virulent progression through unknown mechanisms.

Until recently, little is known about the relationship of *MAT* locus to virulence in fungi. The mating process in *Cryptococcus neoformans* is accomplished by several pheromone response pathways that require genes such as *GPA1*, *STE4*, *STE20*, *STE12*, *STE2* besides pheromone genes^37, 44^. Studies to establish mating events to virulence in *C*. *neoformans* demonstrated that the disruption of *GPA1* led to a sterile and avirulent phenotype due to the deficiency of two major virulence factors, melanin production and capsule formation^44^. In addition, the *MATα* allele, *STE12α* of *C*. *neoformans*, was found to affect both capsule and melanin production, and further was required for monokaryotic fruiting^45^. Moreover, while the major cytoplasmic 5′** → **3′ exonuclease Xrn1p in *C*. *neoformans* is responsible for uni- and bisexual mating, it has been linked to virulence associated phenotypes such as growth at 37 °C, capsule and melanin^46^.

In our study, *M*. *irregularis* isolates of opposite mating types, though based on limited isolate from each mating type, had elicited different levels of immune response, suggested that the virulence between them could be different. How could these mating events influence the virulence in *M*. *irregularis* remain to be unraveled.

Hitherto, the innate sensing of Mucorales spp. by immune and/or nonimmune (e.g. endothelial) cells and the role of adaptive immunity in patients with mucormycosis are still poorly understood. Blakeslee reported that in many species of Mucorales the (+) mating type regularly showed greater vigor than its (−) homolog, and he hypothesized that an unidentified factor associated with the (+) mating type probably exists during the process of zygospore germination, which asserts a dominance over the (−) components^47^. Of interest, *β*-glucan exposure during germinating growth of *Rhizopus* triggered dectin-1 signaling in human dendritic cells and resulted in robust induction of the IL-23/Th-17 responses^48^. Also the mannan could inhibit the macrophage-mediated phagocytosis of conidia and inhibit the cell-mediated immune response^49^. These finding led us to investigate that whether the cell wall of different mating type isolates possessed varying contents of polysaccharide such as *β*-glucan or mannan which might elicit different levels of immune responses. In addition, we observed that the colonies and sporangiospores of *M*. *irregularis* B50k /r (+) were more grayish than CBS103.93 (−) (see Supplementary Fig. S4), but the mutant B50n was more white than the latter (see Supplementary Fig. S4), and the colonies of (−) type isolates had color variation. Therefore, the pigment which has long been recognized as virulence factor in many fungi^45^, maybe not a case for *M*. *irregularis*. The dissimilarity between *SexP* and *SexM*, suggested that these genes might have evolved to regulate different set of genes including virulent factors or being regulated differently. Since the virulence of a pathogen is associated polygenic traits, these mating coordinated cellular events are needed to be studied in *M*. *irregularis*, and to test whether other mating associated genes such as the homologue of *Xrn1p* in *M*. *irregularis* could also affect the virulence.

Although (+) mating type appeared to be more virulent, (−) mating type was more prevalent in our collection; 14 out of 17 clinical isolates had (−) mating type. This skewed mating ratio raised questions on the disease transmission mechanism and the role of the *SexM* on *M*. *irregularis* virulence as well. We would not have a good explanation until additional sampling and thorough investigation on the favourable environment and growth requirement for each mating type are determined. Up date, only ~30 cases so far have been reported worldwide, and both isolates outside of Asia, which reported by Schell’s group were happened to be SexP^12^ (as shown in Supplementary Table S1). Thus, the mechanisms of pathogenesis of *M*. *irregularis* require more in-depth investigation. Nevertheless, our conclusion are also reasonable as the mating type bias is not a phenomenon confined solely to *M*. *irregularis* isolates, although equal frequency of mating types could be the norm across the majority of studies^50^. And the mating type skew had already been found in another member of Mucorales. In *M*. *piriformis*, which causes pear rot, the two mating types occurred in approximately equal proportions in one half of the orchards sampled, but the (+) mating type predominated in the other half^29^. Besides, Kano^51^ suggested that human pathogenic fungi were biased toward single mating type. For example, the ratio of MAT1-1: MAT1-2 of *Sporothrix globosa* which isolated from different area in Japan was 1:3^51^. Moreover, clinical and environmental isolates of *C*. *neoformans* display a severe bias of the MATα mating-type over MAT**a** (the ratio was ca. 45:1 in environmental isolates and ca. 30:1 in clinical isolates)^39^. Nieuwenhuis concluded that such a skew is consistent with rare sexual reproduction and/or strong selection on pleiotropic effects of a mating-type allele^50^.

Other than the possible geographic preference, this mating type bias in our study may be related to virulence itself. As demonstrated in the murine model, mating type (+) induced a stronger inflammatory and cytokine response than (−) mating type strain. For control of fungal infection, during immune responses, IL-17 is responsible for recruiting neutrophils and to synthesize antimicrobial peptides and proinflammatory cytokines, and IL-17 producing T cells can shift to produce IFN-γ and induce Th-1 responses^52^. Also, natural killer (NK) cells exhibited anti-infective activity against *R*. *oryzae*, which is the most virulent mucormycete^53^. In addition, a previous study showed that *Pseudomonas aeruginosa* could escapes cytotoxic T lymphocytes response during chronic infection^54^. Thus, this facts led us to speculate that in *M*. *irregularis*, SexP (+) isolates with higher virulence might be destroyed by these immunocompetent cells, in contrast, the SexM (−) isolates which induced a weak immune response might partial escape from CD4^+^ cytotoxic T lymphocytes but persistently present as antigen, eventually appearing as chronic infection. This may explain the preference of a chronic infected model of *M*. *irregularis* on the skin and a longer duration of infection in clinical cases, but we cannot rule out other mechanisms, of which (−) mating type may contribute to virulence on the skin. To answer this question, more isolates from China and the world are required to understand their genetic diversity, mating type, and virulence activities.

In conclusion, we have characterized, for the first time, the two different mating types (SexM and SexP) in *M*. *irregulairs*, and confirmed that the clinical isolates from China were predominantly SexM in *MAT* locus. We discovered a natural mutant with multiple deletion/mutation in *MAT* locus and lost certain capacity to mating, indicating the role of *MAT* locus in the regulation of mating. Also, from murine model, we showed that *M*. *irregularis* infection caused lesion and disease proliferation in spleen and induced production of inflammasome-dependent cytokines at 15 DPI. Moreover, there were differential responses of cytokines towards the two mating types in *M*. *irregularis*. The SexP (+) type appeared to be more virulent than the SexM (−) mating type. The future is to further understand the genetic variability of the isolates, the molecular mechanism of sex and its gene regulation in depth to gain further insights into their impact of infection and virulence.

## Materials and Methods

### Fungal isolates and growth conditions

A total of 17 *M*. *irregularis* clinical isolates collected from different regions of China (CBS103.93 and CMFCCC B50e~B50t, see Supplementary Table S1) were *ex situ* conserved at Centre of Medical Fungi, China Committee for Culture Collection of Microorganisms (CMFCCC), Nanjing, China. Specifically, *M*. *irregularis* CBS103.93 was the first reported isolate in China from a primary cutaneous infection in a 34-year-old farmer suffered from a skin lesion for about 17 years^2^. B50k was isolated from a 64-year-old man who was diagnosed recently by our team (unpublished data), based upon a 3-year-history of a cutaneous lesion on right limb (see Supplementary Fig. S1). For sporangiospore production, all isolates were grown on malt extract agar (MEA) (Oxoid, UK) at 28 °C. DNA extraction was performed from each MEA culture that was inoculated with 1000 spores onto a sheet of cellophane.

### Mating type crosses

The lengths and widths of 20 randomly chosen sporangiospores in each isolate were measured. Crossing combination was performed by pairing the mycelial agar plug (5 mm × 5 mm) of reference strain CBS103.93 (−) with each of other *M*. *irregularis* isolates on MEA, followed by incubation in the dark at 25 °C. The presence of zygospores suggests the pairing isolate having the opposite mating type to the strain CBS103.93 (−). Additional crosses experiments were performed by pairing B50k, which was characterized to be (+) type, and B50n, which was determined to be (+/0) type with each of other isolates as mentioned above. Each pairing had three replicates, and the whole crossing combination experiment was repeated twice.

### Morphological analyses

The lengths and widths of 20 randomly chosen sporangiospores in each isolate were measured. For scanning electron microscopy (SEM), the sporangiospores and zygospores of *M*. *irregularis* were collected and suspended in 0.1 M sodium cacodylate, then immobilized on Millipore Nitrocellulose filters (Millipore HAWP, 0.46 μm), which were immediately fixed in 2% glutaraldehyde, 0.05% malachite green oxalate in 0.1 M sodium cacodylate buffer. The fixation buffer was then removed and the membrane was washed in 0.1 M sodium cacodylate prior to being subjected to an ethanol dehydration series (2 times for 10–15 min in 25%, 50%, 75%, 95%, and 3 times in 100% ethanol). Samples were then critical point dried, sputter coated, and imaged with the Hitachi S3000N (Hitachi Company, Japan) at Nanjing agricultural University.

### Characterization and molecular analysis of *MAT* in *M*. *irregularis*

To obtain the putative *MAT* locus (sex) genes in *M*. *irregularis*, the published sequences of the *SexM/P* genes in *Phycomyces blakesleeanus* (EU009461, EU009462), *M*. *circinelloides* (FJ009106, FJ009107 and HM565940), *Rhizopus oryzae* (HQ450316) and *M*. *mucedo* (JN587498) from NCBI were used as query sequences to search against the assembled *M*. *irregularis* genome (B50p, accession No. AZYI00000000.1) by BLASTP and TBLASTN programs (e-value < 1e-10). Then the *MAT* locus sequence derived from the genomic analysis of B50p was used as a template for primer design to target the *SexM/P* gene loci in 17 tested isolates. Specific primers were designed targeting between the 5’ of *tpt* and the 5’ of RNA helicase using MacVector 11 (Accelrys, USA) (listed in Supplementary Table S2).

The genomic DNA of the 17 isolates’ was extracted as previously described^15^. The PCR mix contained (25 μl total volume): 12.5 ng genomic DNA, 10 pmol primers, 12.5 μl 2 × Vazyme LAmp Master Mix (P312, Vazyme, China). The temperature profile was: 94 °C for 3 min, 35 cycles of 94 °C for 30 s, 55 °C for 30 s, and 72 °C for 3 min. The amplified fragments were isolated from an gel purified and sequenced. FGENESH^55^ was used to predict the putative transcription start site (TSS) and number of exons. The sense of genes in the *MAT* locus was determined in ORF Finder (https://www.ncbi.nlm.nih.gov/orffinder/) and displayed with a dot plot diagram. Finally, the ORF of *SexM/P* was cloned using gene specific primers (listed in Supplementary Table S2), sequenced and verified in each tested isolate.

The sequences of *MAT* genes from 17 *M*. *irregularis* isolates were aligned using MacVector 11 with manual adjustment. The phylogenetic relationship among these isolations was inferred with MEGA 6.0^56^ using neighbor-joining (NJ) algorithms with the following parameters: P distance, pairwise deletion and bootstrap (1,000 replicates). Also the amino acid sequences of SexM/P were aligned with SexM/P sequences reported from other Mucorales species (listed in Supplementary Table S3). A phylogenetic tree was constructed by NJ with 500 bootstrap replications using MEGA 6.0 as described above. These newly determined *MAT* sequences of *M*. *irregularis* isolates have been deposited in GenBank under accession numbers KY434081 to KY434097.

### *M*. *irregularis* mouse infection model

Seven-week old BALB/c female mice were housed at 22 °C under a 12-h light-dark cycle and with *ad libitum* access to food and water. This study was carried out in strict accordance with the recommendations in the guide for the care and use of laboratory animals by the authority of the People’s Republic of China. The experimental protocols were reviewed and approved by the Medical Ethics Committee in the Chinese Academy of Medical Sciences and Peking Union Medical College (Permit Number: 2016-018). The mice were assigned to five groups (n = 20/per group) which were infected by CBS103.93 (−), B50k (+), B50n (+/0), *Rhizopus oryzae* B81a (as the positive control), and the saline (0.9% NaCl as the negative control), respectively. *R*. *oryzae* B81a was also obtained from CMFCCC and cultured on MEA for seven days at 28 °C before the experiment. There was no significant difference in mice gender distribution and weight among groups.

Spores of the respective isolates were harvested by washing the agar surface with sterile saline containing 0.05% Tween 80. The spore suspension was filtered through nylon filters (11 μm pore size), counted on a hemocytometer and re-suspended with saline. An aliquot of 10^6^ spores/0.1 mL was injected into each mouse via intradermal inoculation into the side near a flank of the back.

Mice were monitored twice daily for any signs of illness for 15 days, and the body weight was monitored. The size of lesion occurrence was observed in four infected groups of mice respectively. Fifteen days after the injection, the mice were sacrificed and the spleens, kidneys and livers were excised from each mouse. The organs were weighed after excluding the adipose tissue. Organ coefficient was calculated as the percent of the ratio of the wet weight of the organ to the body weight.

### Hematoxylin-eosin (HE) and periodic acid-Schiff (PAS) staining

Biopsies were obtained from infected mice on 15 days post-infection (DPI). Tissues were fixed in 10% buffered formalin and embedded in paraffin. The slides were stained with HE and PAS to identify fungal elements to observe the changes in the skin layers and subcutaneous tissues.

### Serum cytokine measurement

Blood samples were also collected from mice at 15 DPI. Serum samples were obtained after centrifugation (2000 g for 10 min) of nonheparinized whole blood to remove the blood clots. Thirty-six cytokines including interferon-γ (IFN-γ), IL-17, IL-22, IL-4 were measured in the serum with Luminex using ProcartaPlex multiplex luminex immunoassays panels, Affymetrix (eBioscience, Waltham, USA) according to the manufacturer’s instructions in a Luminex 100/200 system (Luminex, Austin, USA). Results from technical duplicates were averaged and indicated as Mean ± SD.

### Flow cytometric analysis

Fresh spleens were harvested from each group, then placed in 35-mm dishes containing RPMI 1640 medium with 10% Foetal bovine serum (FBS), and homogenized by smashing with the plunger from a 10-ml syringe. The homogenate was passed through a 70 μm cell strainer, and the erythrocytes were lysed with RBC Lysis Buffer (BioGems international, Westlake Village, USA). The remaining cells were counted using TC20 Automated Cell Counter (Bio-Rad, Hercules, USA) and centrifuged (1200 g for 8 min), and then were stained with PE-conjugated CD4 antibodies (eBiosciences) for 30 min at 4 **°**C. Cells stained with the appropriate isotype-matched immunoglobulin were used as negative controls. The cells were fixed and permeabilized using a Cytofix/Cytoperm kit (BD Biosciences, Sparks, MD) according to the manufacturer’s instructions. Intracellular IL-17A, IL-4 and IFN-γ were detected using APC-conjugated antibodies (eBiosciences) in a permeation buffer. The cells were analyzed using flow cytometer (Attune NxT acoustic focusing cytometer, Thermo Fisher Scientific, Waltham, USA).

### Statistics

All experiments were repeated three times. To analyze the differences among the groups, one-way ANOVA followed by Tukey’s multiple comparison test was utilized using statistical software (GraphPad Prism Software, version 4.03, USA). The data in the graphs were expressed as the mean values ± SD; *p < 0.05, **p < 0.01, and ***p < 0.001 was considered statistically significant.

## Electronic supplementary material

Supplementary information
