# Supplementary material for: The influence of the mating type on virulence of Mucor irregularis
Source: Sci Rep. 2017 Sep 6;7:10629. doi: 10.1038/s41598-017-10954-2 (PMC5587739; doi:10.1038/s41598-017-10954-2)
Supplement: Supplementary file 1 — Supplementary information [file 41598_2017_10954_MOESM1_ESM.docx]

**The influence of the mating type on virulence of *Mucor irregularis***

Wenqi Xu, Guanzhao Liang, Jingwen Peng, Zhimin Long, Dongmei Li, Meihua Fu, Qiong Wang, Yongnian Shen, Guixia Lv, Huan Mei, Clement K. M. Tsui, Weida Liu

**Supplementary Table S1.** *Mucor irregularis* isolates included in this study.

| Isolate no. | Mating type | Isolate source | Geographic origin | Length | Width | Ref. | |
| --- | --- | --- | --- | --- | --- | --- | --- |
| DUMC 150.04 | + ^1^ | Human, skin | USA, Carolina | 4.4 – 8.8 | 2.5 – 4.4 | | [1] |
| NRRL 32535 | + ^1^ | Human, skin (nasal septum) | India | 6.0 – 8.5 | 2.5 – 4.5 | | [2] |
| CBS 103.93 | – ^1,2^ | Human, skin (wrist) | China, Jiangsu | 5.6 – 12.8 | 4.3 – 10.3 | | [3] |
| B50e | – ^1,2^ | Human, skin (face) | China, Shanghai | 5.3 – 9.5 | 3.4 – 6.4 | | [4] |
| B50f | – ^1,2^ | Human, skin (face) | China, Shandong | 6.4 – 11.7 | 3.5 – 8.0 | |  |
| B50g | – ^1,2^ | Human, skin (face) | China, Hubei | 3.7 – 8.3 | 2.9 – 5.7 | | [5] |
| B50h | – ^1,2^ | Human, skin (face) | China, Anhui | 5.5 – 9.6 | 3.9 – 6.6 | | [6] |
| B50i | – ^1,2^ | Human, skin (face) | China, Jiangsu | 5.1 – 9.4 | 3.2 – 5.3 | | [7] |
| B50j | – ^1,2^ | Human, skin (right upper extremity) | China, Jiangsu | 4.7 – 11.5 | 3.5 – 6.2 | | [8] |
| B50k | + ^1,2^ | Human, skin (upper extremity) | China, Jiangsu | 6.1 – 13.2 | 3.7 – 9.2 | |  |
| B50l | – ^1,2^ | Human, skin | China, Hubei | 4.8 – 11.2 | 3.3 – 8.0 | |  |
| B50m | – ^1,2^ | Human, skin (face) | China, Guangdong | 5.8 – 9.6 | 3.6 – 6.9 | | [9] |
| B50n | +/0 ^1,2^ | Human, skin (face) | China, Sichuan | 5.1 – 11.6 | 2.0 – 6.5 | | [10] |
| B50o | – ^1,2^ | Human, skin (face) | China, Sichuan | 4.8 – 13.3 | 3.2 – 8.9 | | [11] |
| B50p | – ^1,2^ | Human, skin (face) | China, Chongqing | 4.0 – 10.7 | 3.9 – 5.8 | | [12] |
| B50q | – ^1,2^ | Human, skin (face) | China, Jiangsu | 6.3 – 11.6 | 2.8 – 6.1 | |  |
| B50r | + ^1,2^ | Human, skin (upper extremity) | China, Shandong | 3.8 – 10.5 | 3.2 – 7.1 | | [13] |
| B50s | – ^1,2^ | Human, skin (face) | China, Shandong | 5.0 – 12.6 | 3.2 – 6.4 | | [14] |
| B50t | – ^1,2^ | Human, skin (the dorsum of left hand) | China, Jiangxi | 5.1 – 13.3 | 3.2 – 7.7 | | [15] |

^1^determined in mating assays.

^2^determined by sequencing the sex locus.

References

1. Schell, W.A., O’Donnell, K. & Alspaugh, J.A. Heterothallic mating in *Mucor irregularis* and first isolate of the species outside of Asia. *Med Mycol.* 49, 714-23 (2011).

2. Hemashettar, B.M. *et al*. Chronic rhinofacial mucormycosis caused by *Mucor irregularis* (*Rhizomucor variabilis*) in India. *J. Clin Microbiol*. **49**, 2372-5 (2011).

3. Zheng, R. & Chen, G. A non-thermophilic *Rhizomucor* causing human primary cutaneous mucormycosis. *Mycosystema* **4**, 45-57 (1991).

4. Zhao, Y. *et al*. Primary cutaneous mucormycosis caused by *Rhizomucor variabilis* in an immunocompetent patient. *Mycopathologia*. **168**, 243-7 (2009).

5. Hu, Z.M. *et al*. Primary cutaneous mucormycosis due to *Rhizomucor variabilis*: a case report. *Chinese Journal of Dermatology* **43**, 259-262 (2010). [In Chinese.]

6. Zhang, S.P. *et al*. A case of facial cutaneous mucormycosis due to *Rhizomucor variabilis*. *Chinese Journal of Dermatology* **43**, 134 (2010). [In Chinese.]

7. Lu, XL. *et al*. Primary cutaneous zygomycosis caused by *Rhizomucor variabilis*: a new endemic zygomycosis? A case report and review of 6 cases reported from China. *Clin Infect Dis.* **1**, 49(3):e39-43 (2009).

8. Qi, B. *et al*. Primary cutaneous zygomycosis caused by *Rhizomucor variabilis*: a case report and literature review. *Journal of Clinical Dermatology* **41**, 329-332 (2012). [In Chinese.]

9. Qin, W. *et al*. A case report of primary cutaneous mucormycosis caused by *Rhizomucor variabilis*. *Diagnosis and Therapy Journal of Dermatovenereology* **17**, 14-17 (2010). [In Chinese.]

10. Jiang, X., Wang, S., Zhao, T.J. & Ran, Y.P. A case of cutaneous mucormycosis. *Journal of Clinical Dermatology* **32**, 271 (2003). [In Chinese.]

11. Ran, Y.P. *et al*. Rhinocerebral mucormycosis after the operation of turbinectomy: a case report. *Chinese Journal of Mycology* **1**, 28-30 (2006). [In Chinese.]

12. Zhou, C.J. *et al*. A case of primary cutaneous mucormycosis caused by *Rhizomucor variabilis*. *Chinese Journal of Mycology* **6**, 361-362 (2011). [In Chinese.]

13. Li, C.Y., Li, Y. & Hu, Z.M. A case report of primary cutaneous mucormycosis caused by *Rhizomucor variabilis*. *Journal of Clinical Dermatology* **33**, 158-159 (2004). [In Chinese.]

14. Li, C.Y., Xu, Y.H. & Hu, Q.F. Facial cutaneous Rhizomucormycosis caused by *Rhizomucor variabilis*: a case report. *Chinese Journal of Mycology* **1**, 284-285 (2006). [In Chinese.]

15. Xia, X.J., Shen, H. & Liu, Z.H. Primary cutaneous mucormycosis caused by *Mucor irregularis*. *Clin Exp Dermatol*. 40, 875-8 (2015).

**Supplementary Table S2.** Sequences of PCR primers used in the present study.

| Primer Name | Sequence |
| --- | --- |
| MAT locus forward | AATGTTGACTTCCAGGTATCCGAC |
| MAT locus reverse | GCCTCGTTTTCACTTTCTACTACGG |
| *SexP* forward | GCCCAACCAATAGCAATAG |
| *SexP* reverse | CTGGACAGCGGAGTTTTAT |
| *SexM* forward | CATTCCTCCTCTTTCTTTCG |
| *SexM* reverse | CGATTGCGGTTGTTGGACAT |

**Supplementary Table S3.** SexM/P homologus proteins in other Mucorales.

| Protein | Strain | GenBank ID |
| --- | --- | --- |
| plus sex HMG-domain protein | *Rhizopus delemar* | ADT91595.1 |
| SexP | *Rhizopus oryzae* | ADU04732.1 |
| SexP | *Mucor mucedo* | AFA26127.1 |
| HMG protein | *Mucor circinelloides* | ADR69862.1 |
| MAM1_0357c09999 | *Mucor ambiguus* | GAN10458.1 |
| SexP | *Phycomyces blakesleeanus* | ABX27914.1 |
| SexP | *Syzygites megalocarpus* | AET35404.1 |
| SexP | *Blakeslea trispora* | CDN67530.1 |
| SexP | *Phycomyces nitens* | CED81984.1 |
| SexM | *Rhizopus oryzae* | ADT91545.1 |
| SexM | *Rhizopus delemar* | ADU02298.1 |
| SexM | *Mucor mucedo* | AFA26123.1 |
| SexM | *Mucor circinelloides* | ADR32119.1 |
| SexM | *Phycomyces blakesleeanus* | ABX27909.1 |
| SexM | *Syzygites megalocarpus* | AET35419.1 |
| SexM | *Blakeslea trispora* | CDN67533.1 |
| SexM | *Phycomyces nitens* | CED81987.1 |
| SexM | *Lichtheimia corymbifera* | CDH60400.1 |

**Supplementary Fig. S1.** Clinical comparison of lesions caused by *Mucor irregularis* CBS103.93 and CMFCCC B50k. A. Right hand of the patient showing symptoms of the mucormycosis caused by CBS103.93^2^. B. Erythema, plaques, ulcers, and some black scab on the surface of the patient’s right [upper](javascript:void(0);) [limb](javascript:void(0);) infected by B50k. In brief, the infection course and clinical symptoms vary with each case such as the duration (17 years and 3 years, respectively) and manifestations (local plaque and expanding erythema, plaques, ulcers, respectively).

SexP

SexM

**Supplementary Fig. S2.** Phylogenetic tree based on the predicted SexM and SexP proteins. Sequences of the SexM and SexP proteins of *Mucor irregularis* and other Mucorales which were downloaded from GenBank (listed in Supplementary Table S3) were aligned and analyzed by Neighbor-joining analysis. The phylogenetic tree was constructed by Neighbour-Joining with 500 bootstrap replicates using MEGA 6, bootstrap support values are shown by the numbers near the branches (values ≥ 60).

**Supplementary Fig. S3.** Phylograms of 17 isolates of *Mucor irregularis* from China based on MAT locus by Neighbor-Joining method implemented in MEGA 6 software. Bootstrap values (>60), based on 1000 replicates of the Neighbor-Joining analysis, are shown on branches.


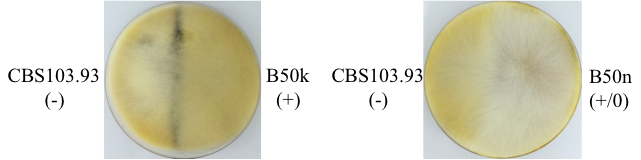


**Supplementary Fig. S4.** The mating reactions of *Mucor irregularis* CBS103.93 with B50k and B50n.
